# Investigating acoustic startle habituation and prepulse inhibition with silent functional MRI and electromyography in young, healthy adults

**DOI:** 10.3389/fnhum.2024.1436156

**Published:** 2024-08-12

**Authors:** Laura F. Naysmith, Owen O’Daly, Ana Beatriz Solana, Florian Wiesinger, Simon Hill, Steven C. R. Williams, Veena Kumari

**Affiliations:** ^1^Centre for Neuroimaging Sciences, Institute of Psychiatry, Psychology and Neuroscience, King’s College London, London, United Kingdom; ^2^GE Healthcare, Munich, Germany; ^3^Centre for Cognitive and Clinical Neuroscience, College of Health, Medicine and Life Sciences, Brunel University London, London, United Kingdom; ^4^Department of Psychology, Institute of Psychiatry, Psychology and Neuroscience, King’s College London, London, United Kingdom

**Keywords:** acoustic startle reflex, startle modulation, prepulse inhibition, startle habituation, functional magnetic resonance imaging, electromyography

## Abstract

**Introduction:**

Startle habituation and prepulse inhibition (PPI) are distinct measures of different sensory information processes, yet both result in the attenuation of the startle reflex. Identifying startle habituation and PPI neural mechanisms in humans has mostly evolved from acoustic-focused rodent models. Human functional magnetic resonance imaging (fMRI) studies have used tactile startle paradigms to avoid the confounding effects of gradient-related acoustic noise on auditory paradigms and blood-oxygen-level-dependent (BOLD) measures. This study aimed to examine the neurofunctional basis of acoustic startle habituation and PPI in humans with silent fMRI.

**Methods:**

Using silent fMRI and simultaneous electromyography (EMG) to measure startle, the neural correlates of acoustic short-term startle habituation and PPI [stimulus onset asynchronies (SOA) of 60 ms and 120 ms] were investigated in 42 healthy adults (28 females). To derive stronger inferences about brain-behaviour correlations at the group-level, models included EMG-assessed measures of startle habituation (regression slope) or PPI (percentage) as a covariate. A linear temporal modulator was modelled at the individual-level to characterise functional changes in neural activity during startle habituation.

**Results:**

Over time, participants showed a decrease in startle response (habituation), accompanied by decreasing thalamic, striatal, insula, and brainstem activity. Startle habituation was associated with the linear temporal modulation of BOLD response amplitude in several regions, with thalamus, insula, and parietal lobe activity decreasing over time, and frontal lobe, dorsal striatum, and posterior cingulate activity increasing over time. The paradigm yielded a small amount of PPI (9–13%). No significant neural activity for PPI was detected.

**Discussion:**

Startle habituation was associated with the thalamus, putamen, insula, and brainstem, and with linear BOLD response modulation in thalamic, striatal, insula, parietal, frontal, and posterior cingulate regions. These findings provide insight into the mediation and functional basis of the acoustic primary startle circuit. Instead, whilst reduced compared to conventional MRI, scanner noise may have disrupted prepulse detection and processing, resulting in low PPI and impacting our ability to map its neural signatures. Our findings encourage optimisation of the MRI environment for acoustic PPI-based investigations in humans. Combining EMG and functional neuroimaging methods shows promise for mapping short-term startle habituation in healthy and clinical populations.

## 1 Introduction

The startle reflex is an involuntary motor response to a sudden and intense sensory stimulus, and it is thought to represent a defensive mechanism (Graham, 1975). This reflex response is consistently observed across mammals, followed by arousal of the sympathetic nervous system (Yeomans et al., 2002). Startle habituation and prepulse inhibition (PPI) are two forms of startle plasticity which result in the attenuation of the startle reflex response; however, these two processes reflect different information processing mechanisms (Geyer and Braff, 1982; review, Swerdlow et al., 2016). Startle habituation occurs in response to a formerly novel startling stimulus on repeated presentation (Thompson and Spencer, 1966). It is a simple form of non-associative learning and reflects the redundancy of behavioural relevance of the stimulus over time (review, Rankin, 2009) occurring within a session (short-term habituation) and between sessions (long-term habituation) (Wang, 1993). PPI, on the other hand, is induced by prepulse-pulse pairing of sensory stimuli (Graham, 1975) with short intervals (Oranje et al., 2006) and is expected to occur on first presentation. It is considered an operational measure of sensorimotor gating which, according to some models, serves as the protection of information processing, i.e., the prepulse filters the pulse leading to reduced startle amplitude (Braff and Geyer, 1990). Clinical studies have shown startle habituation and PPI impairments in Parkinson’s disease (Zoetmulder et al., 2014; Chen et al., 2016), Huntington’s disease (review, McDiarmid et al., 2017), schizophrenia (Geyer and Braff, 1982; Meincke et al., 2004; Hammer et al., 2011; San-Martin et al., 2022), post-traumatic stress disorder (PTSD) (Pineles et al., 2016; Meteran et al., 2019), and schizotypal personality disorder (Cadenhead et al., 1993; Giakoumaki et al., 2020). These clinical impairments may reflect aberrations in attentional and gating mechanisms.

The primary startle pathway, which was first outlined in acoustic-focused rodent models, consists of sensory information projections to the caudal pontine reticular nucleus (PnC) in the brainstem to cranial nerve VII which triggers the startle response. Pharmacological research in animals using acoustic startle paradigms has identified that the startle pathway is modulated by the hippocampus (Heuer et al., 2023), medial prefrontal cortex (Bubser and Koch, 1994; Ellenbroek et al., 1996) basolateral amygdala (Decker et al., 1995; Wan and Swerdlow, 1997), thalamus (Kodsi and Swerdlow, 1997; Wolf et al., 2010), and entopeduncular nucleus during acoustic PPI (Lutjens et al., 2012). Indeed, a cortico-striatal-pallido-thalamic (CSPT) for PPI neural circuitry, which was originally reported from animal studies (Caine et al., 1992), has been corroborated in human functional neuroimaging studies (review, Naysmith et al., 2021).

Neural circuitry involved in startle habituation, however, are not well understood. From acoustic-focused rodent models, it has been suggested that startle habituation occurs through synaptic depression in the PnC (Simons-Weidenmaier et al., 2006), yet its source is disputed. Decerebrate rats have shown short-term startle habituation (Fox, 1979; Leaton et al., 1985), indicating modulation of the primary startle pathway from within the brainstem, yet vermal lesions have produced short-term startle habituation impairments in rats (Leaton and Supple, 1986) which suggests cortical regions modulate acoustic primary startle pathway. Although limited, fMRI studies have identified changes in thalamic activity with tactile startle paradigms (McDowell et al., 2006), and brainstem activity with auditory startle paradigms (Kuhn et al., 2020). Neural networks, including the Default Mode Network (DMN) (Hermann et al., 2020) have also been outlined during acoustic startle habituation with positron emission tomography (PET).

Identifying neural mechanisms underpinning startle habituation and PPI and, consequently, sensory information processing, should aim to build on the popularity of acoustic-focused animal models for better translation between animal and human research. Therefore, fMRI studies investigating acoustic startle habituation and PPI should implement auditory paradigms. However, the logistical challenges of the acoustic properties of fMRI, caused by the rapid switching of gradients, cause great difficulty for auditory experiments, which has led to the development of “fMRI-friendly paradigms” using tactile stimulation to elicit startle (Swerdlow et al., 2016). Moreover, auditory stimuli can evoke blood-oxygen-level-dependent (BOLD) responses in the auditory cortex, with BOLD response increasing with louder noise from gradient pulses (review, Peelle, 2014), and task-evoked BOLD responses, such as during working memory tasks (Tomasi et al., 2005). fMRI studies of acoustic startle modulation have overcome this issue of sound presentation with sparse sampling sequences, although how gradient-related noise during image acquisition affects BOLD response or the startle reflex response is unclear. The development of imaging acquisition sequences designed to reduce gradient noise would significantly improve auditory research in fMRI studies by removing the confounding effect of acoustic noise on the study, within analysis of the BOLD response, and to improve participant experience. In this context, Looping Star, a multi-echo zero echo time (ZTE) near-silent pulse sequence offers a novel approach to fMRI research. Looping Star is a multi-echo ZTE pulse sequence which produces T2* weighted gradient echoes, thus is a near-silent pulse sequence for fMRI research (Wiesinger et al., 2019). Looping Star is a 3D image acquisition sequence which minimises gradient-related acoustic noise by making incremental changes in gradient direction through refocusing the signal and excitation of the signal at the next gradient into a continuous loop (Wiesinger et al., 2019). Looping Star is 0.5 dB above ambient room noise (Dionisio-Parra et al., 2020), making it a desirable option for fMRI research to overcome the challenges from acoustic scanner noise, and has shown good sensitivity to BOLD in an auditory oddball task (Damestani et al., 2021).

The current study acquired simultaneous electromyography (EMG) to capture the startle reflex during scanning with Looping Star (Wiesinger et al., 2019) which looked to map the neural correlates of acoustic short-term startle habituation and PPI, respectively, across a young, healthy adult sample. Unlike our earlier fMRI studies that were conducted using tactile stimuli (Kumari et al., 2003, 2007, 2008), this study used an acoustic startle paradigm which was informed by task parameters used in our previous work (Kumari et al., 2003; Aasen et al., 2005; Naysmith et al., 2022). Investigating acoustic startle modulation in the fMRI environment in this study holds unique significance, particularly as acoustic paradigms to study sensory gating impairments have produced robust findings in disorders such as schizophrenia (Geyer and Braff, 1982; Meincke et al., 2004; Hammer et al., 2011), PTSD (Pineles et al., 2016; Meteran et al., 2019) and schizotypal personality disorder (Cadenhead et al., 1993; Giakoumaki et al., 2020).

We expected to replicate Naysmith et al. (2022), with all participants showing habituation to pulse trials and PPI on SOA 60 ms and 120 ms to elicit a decrease in startle response of ~40%. For mapping startle habituation neural circuitry, we hypothesised that brainstem (Kuhn et al., 2020) and thalamic (McDowell et al., 2006) regions would be observed during repeated presentation of pulse stimuli. Region of interest (ROI) activity (brainstem, thalamus) was expected to decrease with more startle habituation. Moreover, we hypothesised that ROI activity would linearly decrease over the task, and that the magnitude of this linear response decrement would be associated with greater startle habituation. For mapping PPI, we expected to observe neural responses from the hippocampus, thalamus, caudate, putamen, insula, globus pallidus, as previously observed in the neuroimaging literature (review, Naysmith et al., 2021). We hypothesised greater neural activity in these ROI during PPI trials (60 ms, 120 ms), compared to pulse trials.

## 2 Materials and methods

The study was approved by the Psychiatry, Nursing and Midwifery Research ethics committee at King’s College London (HR-19/20-18,771).

### 2.1 Participants

Forty-two participants aged 18–34 years (M = 23.71 years, SD = 3.64) (28 female: M = 23.64 years, SD = 3.49; 14 male: M = 23.86 years, SD = 4.05) took part after meeting inclusion criteria of good health, no hearing loss, no history of neurological/psychiatric illness, and without MRI contradictions. Initially, 52 volunteers were recruited, but five participants (one male, four females) were removed due to excessive movement in the scanner (>1 voxel/ 3 mm). Five subjects (four males and one female) were removed from the analysis for small or non-measurable startle responses (<70% response probability on pulse trials). Prior to data scoring, EMG response to each trial was reviewed, and any trial with evidence of ongoing blinks before stimulus onset were excluded. Scoring criteria were identical to those reported previously (Kumari et al., 2008, 2010).

In the sample, two participants were current smokers, one participant was an ex-smoker, and the remaining 39 participants did not smoke. Hormonal contraception use (*n* = 14 women) and menstrual cycle status were recorded in this sample. Caffeine and nicotine were prohibited on the day of assessment, and alcohol and recreational drug use was prohibited on the evening prior and the day of assessment.

### 2.2 Procedure and paradigm

With participants lying comfortably in the MRI scanner, pneumatic MR-compatible headphones and radiotranslucent MR-compatible electrodes were provided and attached by the experimenter. Participants were told that the experiment was to measure their response to auditory clicks and no instructions were given as to attend or ignore these sounds. Participants were requested to stay relaxed whilst keeping their eyes open and focused on a black cross on a light grey background on the screen. Before the experiment began, they were played an example of the startle probe to ensure EMG was functioning correctly and a startle response was shown.

The acoustic stimuli consisted of a startle ‘pulse’ stimulus (40 ms, white noise, 115 dB) and a prepulse stimulus (20 ms, white noise, 85 dB), as presented in our previous work (Naysmith et al., 2022). The prepulse and pulse stimuli were either presented alone or were coupled to form PPI trials. There were three types of PPI conditions with varying SOA (30 ms, 60 ms, 120 ms). The task included three runs, which were separate scanning series. Per Run, trials consisted of eight pulse trials, eight prepulse trials, and 24 PPI trials (eight PPI trials with 30 ms SOA, eight PPI trials with 60 ms SOA, eight PPI trials with 120 ms SOA). Run 1 consisted of four more pulse trials (trials 1–4) than Run 2 and 3. This was designed to allow participants to acclimatise to the acoustic startle probes which was novel in Run 1. In total, Run 1 consisted of 44 trials, and Run 2 and 3 consisted of 40 trials each. Trials were pseudo-randomly ordered to ensure that no trial type was repeated in a sequence, excluding trials 1–4 in Run 1. All participants were presented Runs 1–3 in the same order. Inter-stimulus intervals ranged from 9 to 21 s (M = 15 s). There was a short interval of 3 minutes between each Run for the radiographer to commence the next sequence/Run.

### 2.3 EMG

A detailed list of all equipment can be found in the Supplementary material. EMG was used to measure the startle reflex as an eyeblink. Two radiotranslucent, pre-gelled, MRI-safe electrodes were applied to the right orbicularis oculi muscle. One electrode was 15 mm below the lateral canthus of the participant’s right eye, the other electrode was 15 mm below and 15 mm medial to the first electrode. Electrodes were placed perpendicular to the axis of the scanner. The ground electrode was placed behind the right ear. Electrodes were attached to the skin surface with adhesive medical tape. MR-safe cables, with insulating barriers (diameter 50 mm) to prevent the electrode cable from touching bare skin, were attached to the electrodes and ran in parallel. The leads connected to the chamber cable, which was also taped to the outside of the MRI head coil. The chamber cable then ran out of the bore and through the connector panel into the control room via the waveguide.

#### 2.3.1 EMG acquisition

The raw signal was acquired in real-time using AcqKnowledge (BIOPAC Systems Inc.). The EMG amplifier had its gain set to high (2000) and applied a band-pass filter (low-pass 500 Hz; high-pass 100 Hz). The EMG amplifier was time-stamped with the MRI scanner to determine the onset of stimuli. EMG activity was continuously recorded with a sampling rate of 10 kHz, which characterised MRI noise during scanning.

#### 2.3.2 EMG signal processing

EMG was captured within the MRI scanner and required additional signal processing which was suited to the frequency spectrum of Looping Star (Dionisio et al., 2018). The full process is detailed in the Supplementary material and is based on the six stages of EMG signal processing by Rose (2011) and Bullock et al. (2021).

### 2.4 fMRI

A 3 T General Electric MR750 Discovery scanner (GE Healthcare, Chicago) with 12-channel head coil was used. To reduce head movement, padding was used within the head coil for comfort. A standard T1-weighted inversion recovery-spoiled gradient echo (IR-SPGR) structural image was acquired in the anterior commissure-posterior commissure (AC-PC) plane with the following acquisition parameters: echo time (TE) = 3.04 ms, repetition time (TR) = 7.35 ms, inversion time (TI) = 400 ms, flip angle (FA) = 11°, slices = 196, and slice thickness = 1.2 mm.

Multi-echo Looping Star images (Wiesinger et al., 2019) were acquired with the same acquisition parameters across runs: TEs = [0 ms, 17.9 ms, 35.8 ms], TR = 2.62 s, FA = 3°, spatial resolution = 3x3x3mm, receiver bandwidth (rBW) = ±31.25 kHz, undersampling factor per volume = 3.5, field of view (FOV) = 19.2 cm. The number of volumes and length of scan differed between the runs (Run 1 = 267 volumes; Run 2 = 242 volumes; Run 3 = 237 volumes).

#### 2.4.1 fMRI pre-processing

Looping Star image reconstruction was based on 3D nearest-neighbour gridding, followed by Fourier transformation and root-sum-of-square coil combination, performed using a MATLAB (The MathWorks Inc, 2021) compiled executable running on the scanner. Echo 1 (TE = 0 ms) was not used for further analyses. An optimal combination of the echoes was used to improve the temporal signal-to-noise ratio (tSNR) (Kundu et al., 2012) (see Supplementary material). The first four volumes were discarded from the echo 2 and echo 3 image time series to assure that steady-state was reached.

For each participant, the functional time series for each run were pre-processed in SPM12.^1^ A standard pre-processing pipeline was used, excluding slice-timing correction as Looping Star is a 3D sequence with no discrete slice time correction (Damestani et al., 2021). Functional images were spatially normalised using unified segmentation, co-registered with the T1 image performed using the second echo of each fMRI series and the same transformation was then used to register the third echo timeseries onto the T1 imaging also. Spatial normalisation parameters were derived by means of unified segmentation of the T1-weighted structural image. The resultant deformation fields were applied to the co-registered functional timeseries to normalise them to Montreal Neurological Institute (MNI) reference space. Functional images were then smoothed with a 6 mm full width at half maximum (FWHM) kernel.

### 2.5 Statistical analysis

#### 2.5.1 EMG

Analysis was conducted using SPSS Statistics (version 26) software package and applied an alpha level for significance testing at *p* < 0.05. Normality of data was tested with a Shapiro–Wilk test to determine use of parametric tests.

##### 2.5.1.1 Calculating startle habituation

Raw startle amplitude was captured on each pulse trial. For each participant, an average startle amplitude was calculated across the pulse trials in each Run (Run 1, Run 2, Run 3). Then, the average startle amplitude response on pulse trials was calculated to produce a group average for Run 1, Run 2, and Run 3. An initial measure of startle habituation was conducted by comparing raw startle amplitude on pulse trials on each Run. Statistically lower startle amplitude on later Runs, compared to Run 1, would indicate startle habituation.

Secondly, startle habituation was quantified as a regression slope per participant using each participant’s linear regression on pulse trials across all three Runs:


$$\left(Y=bX+a\right)$$

*Y* was the amplitude of the eyeblink response and was predicted by the log-transformed trial number (*X*). Each participant’s startle response amplitude on trial 1 in Run 1 was used as the intercept (*a*), and the regression slope (*b*) (i.e., the unstandardised beta coefficient) was the rate of startle habituation. Startle habituation slope values (*b’*) were then calculated for each participant based on Montagu’s (1963) formula:

$$\left(b^{\prime }=b-c\left(a-\bar{a}\right)\right)$$

*c* was the standardised beta coefficient from the linear regression which illustrated changes in the regression slope (*b*) on the intercept (*a*). *ā* was the mean value of startle amplitude on pulse trials, excluding trial 1. A negative startle habituation slope value indicated more habituation, with a larger negative slope value meaning faster habituation to pulse trials. This approach is a desirable measure of startle habituation as *b’* becomes independent of the initial startle amplitude which shows huge variability between participants.

Raw startle amplitude to pulse trials on each Run was not normally distributed (Run 1: W = 0.869, *p* < 0.001; Run 2: W = 0.761, *p* < 0.001; Run 3: W = 0.870, *p* < 0.001). Thus, raw startle amplitude to determine habituation to the pulse trials was examined using a Friedman test.

##### 2.5.1.2 Calculating PPI

To calculate PPI for each participant, raw startle amplitude across each SOA trial on each Run was calculated, and then the average raw startle amplitude was calculated across Run 1–Run 3. PPI was then computed for each participant, separately for each SOA on each Run, this took the average raw startle amplitude and calculated a percentage of change of raw startle amplitude on each PPI SOA trial type, compared to pulse trials. Overall PPI was calculated separately for each SOA across Run 1–Run 3.


$$\left[\frac{\left(a-b\right)}{a}\right]\;x\;100$$

*a* was the startle amplitude over pulse trials and *b* was the startle amplitude over PPI trials. Percentage, rather than absolute amount of PPI (i.e., arithmetic difference between pulse and PPI trials), was used to minimise the influence of individual differences in startle responsiveness. A positive percentage showed a decrease in startle amplitude on the PPI trials, compared to the pulse trials, whereas a negative percentage would indicate an increase in startle amplitude on the PPI trials, compared to pulse trials.

Parametric tests were justified as data did not deviate from normality on 60 ms (W = 0.981, *p* = 0.710) and 120 ms SOA (W = 0.984, *p* = 0.802), however this was not the case for 30 ms SOA (W = 0.942, *p* = 0.032). PPI at each SOA (30 ms SOA, 60 ms, 120 ms) was compared using a repeated measures one-way analysis of variance (ANOVA) to examine PPI across the sample based on SOA. Age effects (Giannopoulos et al., 2022; de Oliveira et al., 2023), sex effects (Aasen et al., 2005; Naysmith et al., 2022), menstrual cycle phase (Jovanovic et al., 2004) and the influence of startle habituation (Blumenthal, 1997) were controlled, as they have been shown to affect PPI. Smoking effects have also been observed on PPI (Della Casa et al., 1998), thus PPI at each SOA (30 ms SOA, 60 ms, 120 ms) was compared using a separate repeated measures one-way ANOVA in the non-smoking sample (*n* = 40). For all ANOVAs described earlier, significant interaction or main effects were followed up by lower order ANOVAs and the analysis of simple main effects using t-tests. Repeated measures with more than two levels employed the Greenhouse–Geisser epsilon (*ɛ*) correction.

##### 2.5.1.3 Test reliability

Test reliability for raw startle response on pulse and PPI trials was dependent on normality of data. Consequently, for eliciting a startle response from the pulse trials in each Run, correlations (Spearman’s rho) with a 95% confidence interval were used, as a parametric test would not be suitable. Positive correlations illustrate a linear relationship between measures, which can be used to reflect reliability. Whereas on each SOA across each Run, test–retest reliability of the startle amplitude on PPI trials was assessed with intraclass correlation coefficient (ICC) estimates and their 95% confidence intervals, which is suitable for normally distributed data. The type of ICC was single rater, and the definition of relationship was consistent.

#### 2.5.2 fMRI

fMRI data were analysed using general linear models (GLM) factorial models (multiple regression) to probe startle habituation neural circuitry, and separate general linear models (GLM) factorial models (multiple regression) to probe PPI neural circuitry. We chose not to analyse PPI with SOA 30 ms in the fMRI analysis due to negligible PPI at this SOA. A one sample t-test showed that PPI did not significantly differ from zero across the whole group on 30 ms SOA PPI condition (*t* (41) = −0.275, *p* = 0.784), whereas SOA 60 ms (*t* (41) = 4.015, *p* < 0.001) and SOA 120 ms (*t* (41) = 2.338, *p* = 0.012) PPI conditions did significantly differ from zero.

##### 2.5.2.1 Whole group analysis of fMRI data

###### 2.5.2.1.1 Modelling fMRI analyses at the individual-level

Participant-specific response maps which were generated by means of a fixed-effects (first-level) analysis. The first-level design matrix modelled the five experimental conditions (pulse trials, prepulse trials, PPI SOA 30 ms trials, PPI SOA 60 ms trials, PPI SOA 120 ms trials) and an implicit control condition of rest (baseline). Realignment parameters (x, y, z, pitch, roll, yaw) were included in first-level models as nuisance covariates associated with head movement. The contrast image [pulse trials > baseline] was designed to explore startle habituation, and the contrast images [PPI SOA 60 ms > pulse trials; PPI SOA 120 ms > pulse trials] were designed to explore PPI with two SOA.

To permit us to test for changes in the amplitude of brain responses to startle cues, a first order temporal modulator for each experimental condition was added to the design matrix at the individual-level. Following parameter estimation, a contrast of parameter estimates for the relevant temporal modulator was generated and taken forward to group-level analysis.

###### 2.5.2.1.2 Modelling fMRI analyses at the group-level

These maps were subsequently taken forward to second-level random-effects modelling under the GLM. For startle habituation analyses, the linear regression model predicted the blood oxygen level dependent (BOLD) signal across the sample with experimental matrix, and group as a nuisance covariate. The two linear regression models for PPI predicted the BOLD response across the whole sample on each contrast with the experimental matrix, and age, sex, menstrual cycle phase and startle habituation regression slope values were included as nuisance covariates. In both cases, sex was a nuisance covariate to control for previously reported sex differences in startle habituation and PPI (Abel et al., 1998; Kofler et al., 2001; Kumari et al., 2004). Age was not a nuisance covariate for startle habituation as previous findings do not report age effects (de Oliveira et al., 2023). Menstrual cycle phase was controlled in the PPI regression models, as previous findings document menstrual-cycle related variability on PPI (Jovanovic et al., 2004; Kumari et al., 2010). Startle habituation has been shown to affect PPI (Blumenthal, 1997), and thus was controlled. In addition, the relationship with startle habituation and PPI EMG-assessed measures were examined with startle habituation regression slope values and PPI values were included as covariates in each fMRI analysis, respectively.

##### 2.5.2.2 ROI and whole brain analyses

A ROI approach was taken using *a priori* defined ROI for the startle habituation analysis: i) thalamus and ii) brainstem, consisting of pons and midbrain, and i) thalamus, caudate, putamen, insula, hippocampus and globus pallidus, and ii) brainstem, consisting of pons and midbrain for the PPI analysis. Here, peak-level correction within a small volume was used (*p*_FWE_ < 0.05). ROI mask details can be found in the Supplementary material. The whole brain analysis was conducted to explore startle habituation-related activity using cluster-based inference to determine significance (*p*_FWE_ < 0.05). This was not conducted for PPI as ROI activity has been established in several human functional neuroimaging studies (review, Naysmith et al., 2021).

## 3 Results

### 3.1 Startle habituation

#### 3.1.1 EMG

The startle habituation slope value across the whole group was −1.36, which significantly differed from 0 (*t* (41) = −3.729, *p* < 0.001, *d* = 0.58) with a moderate effect size. However, raw startle amplitude was lowest on Run 3, compared to Run 1 and 2 (Table 1), yet startle amplitude did not statistically differ between Runs (*χ*^2^ = 3.571, df = 2, *p* = 0.168). This may be associated with spontaneous startle recovery resulting from a two-minute break to begin the next series. Comparisons of raw startle amplitude between Runs would therefore capture the spontaneous startle recovery and affect measures of startle habituation. There were significant and positive correlations between startle amplitude on pulse trials in all three Runs (Run 1 and 2: r = 0.872, df = 41, *p* < 0.001; Run 1 and Run 3: r = 0.859, df = 41, *p* < 0.001; Run 2 and 3: r = 0.912, df = 41, *p* < 0.001) indicating good internal consistency. Correlations were significant after Bonferroni corrections (*p* = 0.016). The 95% confidence intervals ranged from 0.748 to 0.953 across all three correlations.

**Table 1:** Mean (standard error of the mean, SEM) startle amplitudes (mV) on pulse trials, startle habituation slope value, and PPI (percentage).

Measure	Overall mean (SEM)
*Raw startle amplitude [mV]*	
Run 1	2.80 (0.33)
Run 2	2.91 (0.40)
Run 3	2.32 (0.23)
*Startle habituation slope values*	
Whole task	−1.36 (0.37)
PPI [%]	
30 ms SOA	−1.13 (4.17)
60 ms SOA	12.99 (3.23)
120 ms SOA	9.67 (3.99)

#### 3.1.2 fMRI

**Figure 1:**
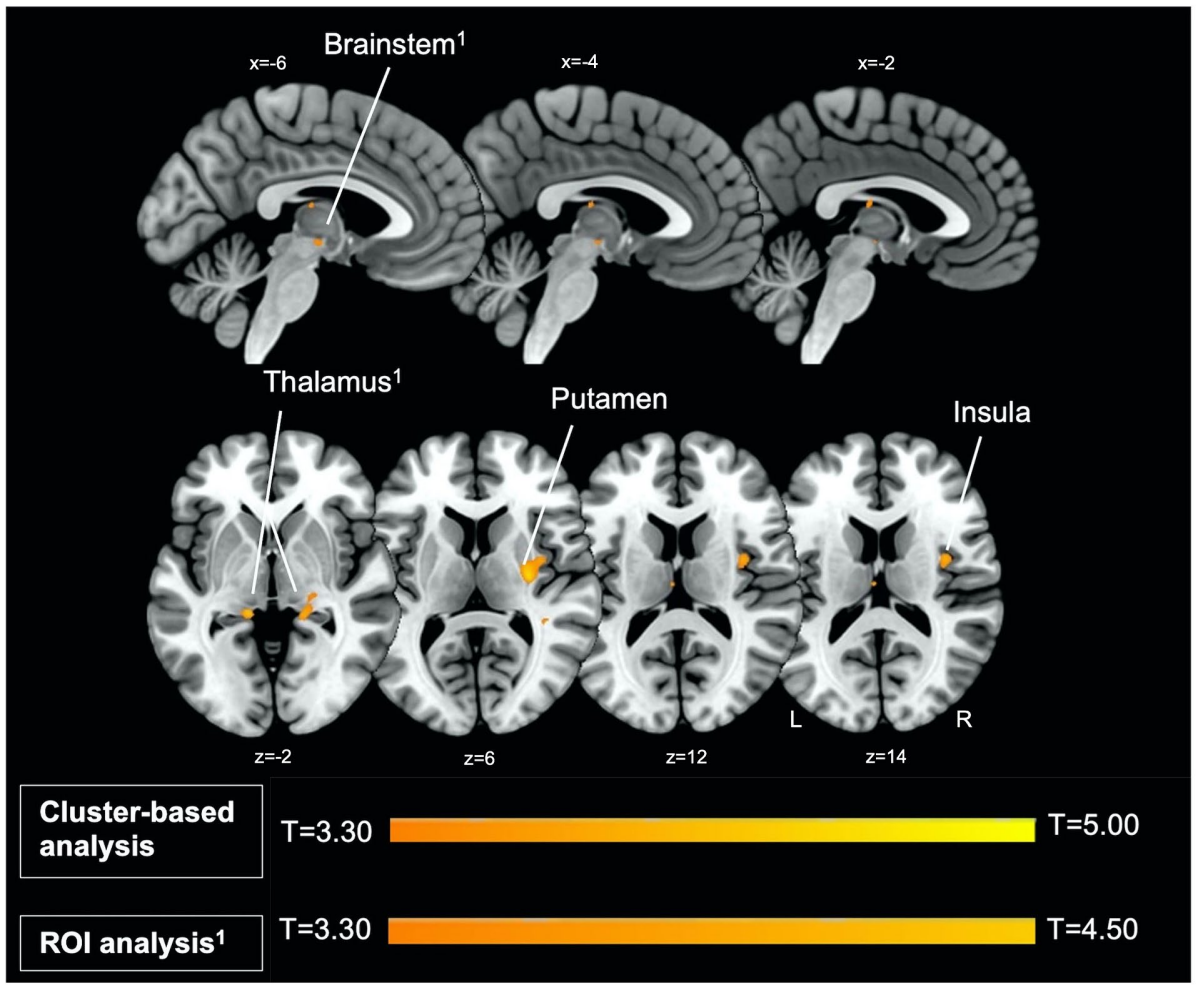
Left parasagittal slices in the lateromedial plane (top) and inferior–superior axial slices (below) show thalamus, brainstem, putamen, and insula regions which comprise the startle habituation neural circuitry. Greater BOLD response in these areas, identified with ROI (thalamus, brainstem) and cluster-based analysis (putamen, insula), was observed during pulse trials, compared to baseline.

**Figure 2:**
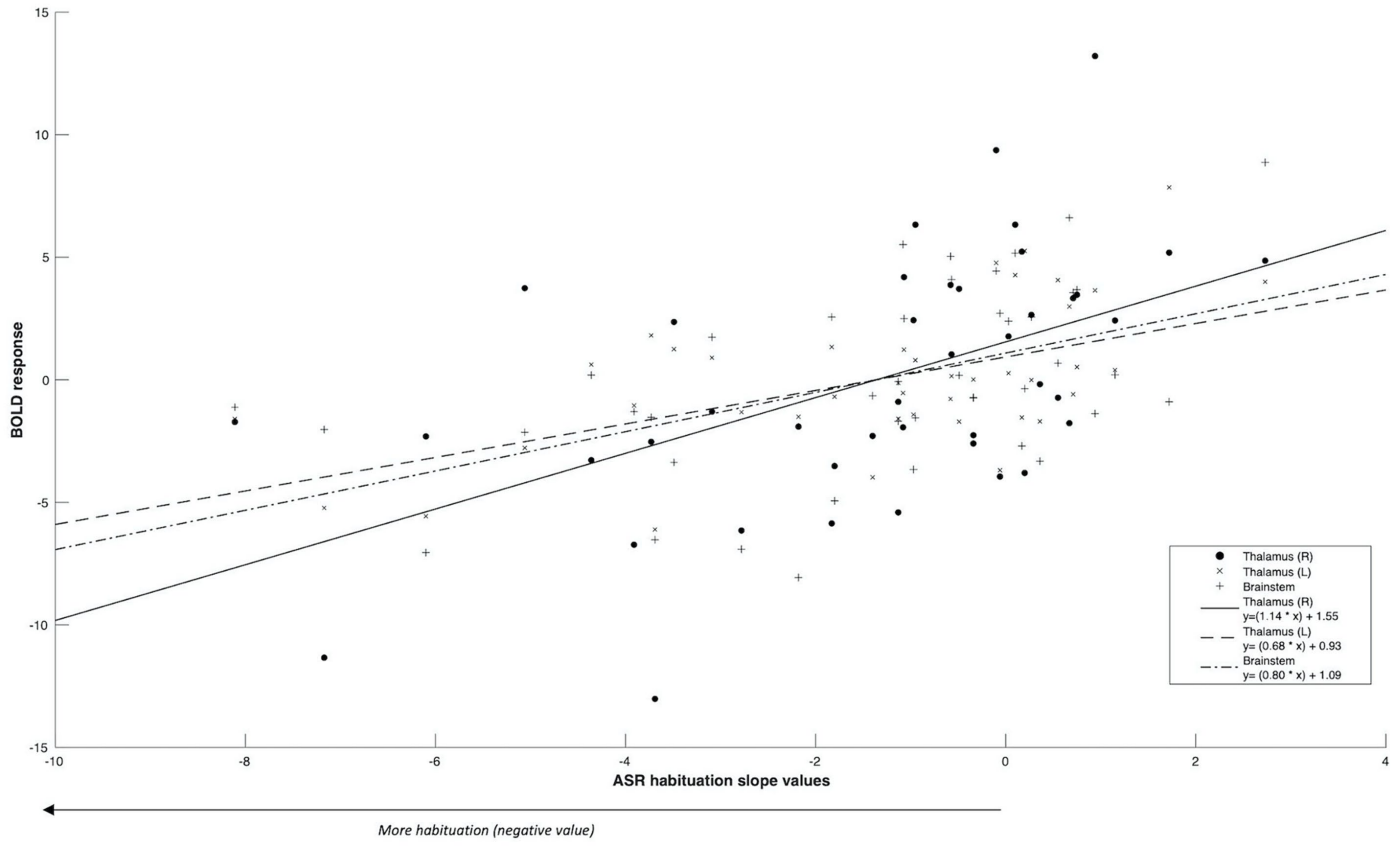
Significant ROI activity correlated positively with startle habituation slope values.

Firstly, we identified significantly greater BOLD activity within the right thalamus (*p*_FWE_ = 0.048) in the startle habituation ROI mask during pulse trials, compared to baseline (Figure 1). This BOLD activity decreased with increasing startle habituation (indicated by negative startle habituation slope values) (Figure 2). In addition, BOLD activity was observed in the brainstem and left thalamus in the startle habituation ROI mask during pulse trials, compared to baseline, but this was not significant after multiple comparisons correction (thalamus: *p*_uncorrected_ < 0.001, *p*_FWE_ = 0.147; brainstem: *p*_uncorrected_ < 0.001, *p*_FWE_ = 0.164); this BOLD activity also positively correlated with startle habituation (Figure 2). Lastly, we observed significantly more BOLD activity in a cluster with a peak in the right putamen, extending to insula, on pulse trials, compared to baseline (*p*_FWE_ = 0.014), and this activity also correlated with startle habituation slope. Table 2 reports all neural activity.

**Table 2:** Neural activity observed during the startle habituation across the whole sample.

Brain region	Hemisphere	MNI coordinates			T	k	*p*_FWE_
		x	y	z			
*ROI analysis*							
Thalamus	R	22	−24	0	4.02	11	0.048
	L	−2	−16	14	3.53	6	0.147^a^
Brainstem	L	−4	−14	−6	3.54	5	0.164^a^
*Whole brain analysis*							
Cluster: putamen, extending to insula	R	30	−12	8	4.65	149	0.014

a Does not survive FWE corrections.

Next, the temporal modulation analysis also showed a significant model fit within the left (*p*_FWE_ = 0.026) and right thalamus (*p*_FWE_ = 0.052) in the startle habituation ROI mask during pulse trials, compared to baseline. Neural responses in two clusters also showed significant changes across time on pulse trials, compared to baseline: the first cluster extending to the right caudate and thalamus (*p*_FWE_ = 0.013), and the second cluster extending to the left caudate, thalamus, cingulate gyrus, and hippocampus (*p*_FWE_ = 0.024). Table 3 reports all neural activity.

**Table 3:** Neural activity observed during the startle habituation using linear temporal modulator across the whole sample.

Brain region	Hemisphere	MNI coordinates			T	k	*p*_FWE_
		x	y	z			
**Changes in BOLD response amplitude across time**							
*ROI analysis*							
Thalamus	R	12	−22	18	3.94	9	0.052
	L	−16	−24	18	4.24	4	0.026
*Whole brain analysis*							
Cluster: caudate and thalamus	R	4	4	8	6.73	181	0.013
Cluster: caudate, thalamus, cingulate gyrus, and hippocampus	L	−14	−26	22	5.22	153	0.024
**Positive association with startle habituation and linear change in BOLD response**							
*ROI analysis*							
Thalamus	R	22	−26	6	4.03	14	0.042
	L	10	−32	6	3.46	2	0.150^a^
*Whole brain analysis*							
Cluster: insula and thalamus	R	30	−26	8	5.24	144	0.029
Cluster: parietal cortex, insula, superior temporal gyrus, hippocampus and caudate	L	−46	−36	20	5.05	531	<0.001
**Negative association with startle habituation and linear change in BOLD response**							
*Whole brain analysis*							
Cluster: frontal lobe extending to middle and inferior frontal gyrus	L	−28	10	38	4.88	320	0.001
Cluster: putamen extending to insula and temporal gyrus	R	28	4	−8	4.14	120	0.052
Cluster: posterior cingulate gyrus extending to occipital gyrus	R	4	−60	10	4.06	135	0.036
Cluster: frontal lobe extending to medial frontal gyrus	L	−12	−20	50	4.19	145	0.029
Cluster: inferior frontal gyrus extending to insula		52	4	10	4.10	101	0.083^a^

a Does not survive FWE corrections.

Across the group, the contrast magnitude (akin to the slope of the change in response amplitude) was significantly associated with startle habituation in the right thalamus (*p*_FWE_ = 0.042) in the startle habituation ROI mask during pulse trials, compared to baseline, but the left thalamus was not significant after multiple corrections (*p*_FWE_ = 0.150). Two clusters, one in the left parietal cortex, insula, superior temporal gyrus, hippocampus and caudate (*p*_FWE_ < 0.001), and the other cluster in the right insula and thalamus (*p*_FWE_ = 0.029) was also significantly associated with startle habituation. Thalamic activity and neural responses in both clusters reflected a decrease in the startle-related BOLD response as the task progressed. Moreover, the whole brain analysis also outlined five clusters during pulse trials, compared to baseline, which correlated with startle habituation and showed the opposite pattern of behaviour across time, whereby a greater increase in neural response amplitude was associated with more startle habituation (indexed by a negative startle habituation slope value). Table 3 reports all neural activity.

### 3.2 PPI

#### 3.2.1 EMG

On average, the 30 ms SOA PPI condition did not elicit a PPI response in this sample, and SOA 60 ms and 120 ms produced a 9–13% decrease in startle response (Table 1). Consequently, there was no significant main effect of SOA (*F* = 0.901, df = 1.611, 59.621, *p* = 0.411, ɛ = 0.806), when controlling for age, sex, menstrual cycle phase, and startle habituation. There were no interactions between SOA and sex (*F* = 0.298, df = 1.611, 59.621, *p* = 0.695, ɛ = 0.806), SOA and age (*F* = 1.228, df = 1.611, 59.621, *p* = 0.293, ɛ = 0.806), SOA and menstrual cycle phase (*F* = 0.799, df = 1.611, 59.621, *p* = 0.430, ɛ = 0.806) or SOA and startle habituation (*F* = 2.235, df = 1.611, 59.621, *p* = 0.295, ɛ = 0.806). ICC estimates indicated excellent reliability in eliciting a PPI response across all three Runs on all three SOA (30 ms: 0.933; 60 ms: 0.960; 120 ms: 0.947), with the 95% confidence intervals ranging from 0.887 to 0.961 on 30 ms, 0.933 to 0.977 on 60 ms, and 0.911 to 0.970 on 120 ms.

When current smokers (n = 2) were removed from the analysis, the 30 ms SOA PPI condition did not elicit a PPI response in this sample, and SOA 60 ms and 120 ms produced a 10–14% decrease in startle response. Again, there was no significant main effect of SOA (*F* = 0.267, df = 1.622, 56.773, *p* = 0.720, ɛ = 0.811), when controlling for age, sex, menstrual cycle phase, and startle habituation, nor any interactions between SOA and sex (*F* = 0.347, df = 1.622, 56.773, *p* = 0.664, ɛ = 0.811), SOA and age (*F* = 1.046, df = 1.622, 56.773, *p* = 0.345, ɛ = 0.811), SOA and startle habituation (*F* = 1.828, df = 1.622, 56.773, *p* = 0.176, ɛ = 0.811), or SOA and menstrual cycle phase (*F* = 0.635, df = 1.622, 56.773, *p* = 0.502, ɛ = 0.81). The analysis revealed that sex (*F* = 9.509, df = 1, 35, *p* = 0.004, ɛ = 0.811) and menstrual cycle phase (*F* = 9.795, df = 1, 35, p = 0.004, ɛ = 0.811) were significant covariates. Bonferroni pairwise comparisons, adjusted for the menstrual cycle phase and sex, revealed significant differences between 30 ms SOA and 60 ms SOA (*p* = 0.010). These findings indicate that, while the overall effect of SOA was not significant, SOA differed significantly when accounting for the menstrual cycle phase and sex. ICC estimates indicated excellent reliability in eliciting a PPI response across all three Runs on all three SOA (30 ms: 0.919; 60 ms: 0.955; 120 ms: 0.949), with the 95% confidence intervals ranging from 0.863 to 0.954 on 30 ms, 0.924 to 0.975 on 60 ms, and 0.913 to 0.971 on 120 ms.

#### 3.2.2 fMRI

No significant neural activity in the PPI ROI mask was observed when analysing data on either contrast [PPI 60 ms SOA > pulse trials; PPI 120 ms SOA > pulse trials].

## 4 Discussion

Building upon acoustic-focused rodent models of startle modulation, this silent fMRI study used Looping Star (Wiesinger et al., 2019) to map the neural correlates of acoustic short-term startle habituation and PPI within healthy young adults. The auditory startle paradigm was presented without the confound of gradient-related acoustic noise associated with fMRI and produced reliable startle responses. We reported startle habituation neural circuitry which was comprised of thalamic, brainstem, striatal, and insula regions, with BOLD response in these regions decreasing with increasing startle habituation. Moreover, we observed changes in thalamic, caudate, cingulate gyrus, and hippocampal BOLD response amplitude across time during pulse trials, compared to baseline. The degree of change in thalamic, insula and parietal BOLD responses correlated with startle habituation, with neural activity decreasing over time with startle habituation. Whereas changes in BOLD response in frontal, dorsal striatal, and posterior cingulate regions increased over time with startle habituation. Combined EMG measures were necessary for providing information on the direction of relationship between fMRI BOLD response during startle habituation, and behavioural changes in acoustic startle response. On the other hand, we did not observe significant neural activity during PPI conditions (60 ms and 120 ms SOA), compared to pulse trials and acoustic PPI was markedly lower in the MRI environment, compared to our previous laboratory-based PPI findings (Naysmith et al., 2022), which may have impacted correlations with anatomical structures.

Firstly, our findings illustrate cortical (insula), subcortical (thalamic, putamen) regions, and the brainstem underpin the neurofunctional basis of short-term startle habituation, which have previously been reported (McDowell et al., 2006; Kuhn et al., 2020). Indeed, modelling startle habituation metrics from simultaneous EMG acquisition captured the direction of the relationship between acoustic startle habituation and neural activity. Furthermore, the study captured a greater linear decrease in thalamic, insula and parietal lobe activity with more startle habituation, revealing an association between the increments or decrements in brain responses across time in these regions and the temporal characteristics of the reflex responses to repeated pulse stimulus. McDowell et al. (2006) suggested that startle habituation may be mediated by the reticular activating system (RAS), as decreasing thalamic and cortical activity may implicate the RAS which projects to other neural substrates via through ascending tracts to the cerebellum, thalamus, cortex, and limbic structures (Yeo et al., 2013) to elicit and inhibit the startle response. Our findings showed a decrease in thalamic activity with decreasing startle amplitude (habituation), which may illustrate a reduced need for sensory processing and relay through the thalamus during startle habituation and implicating the RAS in acoustic short-term startle habituation. On the other hand, Hermann et al. (2020) correlated preserved startle habituation in minimally conscious patients with cortical PET metabolism in large-scale networks including the DMN and salience network. This was thought to reflect cortical mediation of the startle pathway, leading to reduced startle during habituation. The current study observed increasing neural responses over the task in frontal, dorsal striatal, and posterior cingulate regions which correlated with startle habituation. This may illustrate cortical mediation of the primary startle circuitry, possibly in relation to the repeated startle stimuli being perceived as inconsequential and carrying no threat or functional significance in the given context, and thus short-term startle habituation. Overall, our findings may lend support to both cortical and brainstem sources of startle habituation, both of which are associated with the common role of the thalamus and non-specific nuclei projections through the cortex, and/or connectivity between the networks which are linked to volitional behaviour and consciousness (Leon-Dominguez and Leon-Carrion, 2019). Indeed, these theories could not have been explored without illustrating the direction of relationship between BOLD and startle habituation, and the functional changes in neural activity associated with startle habituation using a combined EMG-fMRI approach. Moreover, the application of an acoustic startle paradigm to explore startle habituation would not have been possible in this study without the use of the silent fMRI sequence.

Secondly, we aimed to outline the neural correlates of acoustic PPI, of which our previous tactile PPI research in healthy adult populations identified striatal, thalamic, hippocampal, insula, inferior frontal gyrus and supramarginal gyrus/inferior parietal activity on 120 ms SOA PPI condition (Kumari et al., 2003, 2007). However, we note low PPI (9–13%) across our sample, as previous EMG studies have demonstrated up to an 80% decrease in the startle response in healthy volunteers (Meyhofer et al., 2019; Lei et al., 2021), but no significant neural activity during PPI (60 ms and 120 ms SOA), compared to pulse trials. In this healthy sample, PPI may have been lower than our previous lab-based findings (Naysmith et al., 2022) due to the MRI environment. Similarly, Heidinger et al. (2019) observed a 20% and a 35% decrease in startle response on acoustic PPI trials with 60 ms and 120 ms SOA, respectively, whereas in the mock scanner acoustic PPI was larger (60 ms SOA: 43%; 120 ms SOA: 85%). Schulz-Juergensen et al. (2013) did not observe PPI responses in the healthy or patient population on PPI trials (120 ms and 480 ms SOA) in the MRI, but EMG data collected outside of the MRI in the healthy controls showed a 55% decrease in startle amplitude (120 ms SOA). Although this was a young sample (7–13 years), and maturation of the PPI response may also have produced less PPI.

There may be several reasons to linked with the low PPI in this MRI experiment. Acoustic PPI is influenced by changes in the acoustic environment (Basavaraj and Yan, 2012). The acoustic harmonics of Looping Star may have affected PPI, such as small differences in prepulse (85 dB) to background noise (68 dB to 97 dB) (Blumenthal et al., 2005). In this study, the maximum PPI trials generates is 13%, whereas these same stimuli generated 44% PPI outside of the scanner with a 70 dB noise background in our previous work (Naysmith et al., 2022). The MRI environment may have produced a floor effect on PPI which, consequently, affected correlations with neuroanatomical structures of PPI which have previously been reported. Further research is needed to probe low PPI in this instance and would demonstrate the need for a white noise (70 dB) background in future imaging research, including for silent fMRI sequences.

Finally, the current study demonstrated the utility of combining electrophysiology and fMRI to study startle modulation. In this study, simultaneously acquired EMG data can be used to demonstrate reliability and construct validity of the paradigm, in addition to promoting stronger inferences about brain-behaviour relationship by exploring changes in startle response and the primary startle circuitry (review, Wendt et al., 2023). In this instance, the precise nature of this relationship pairing BOLD responses with startle behaviour (EMG measures and BOLD) is thought to indicate the physiology of startle modulation, meaning that the level of brain activation might influence the magnitude of the startle response, or during PPI, the degree of inhibition caused by the prepulse. This suggests that there might be a continuous influence that modulates the primary startle circuitry, affecting how the brain responds to stimuli and how the BOLD signal is generated, as opposed to changes in BOLD signal associated with the stimulus itself or the motor response to the startle. This research should encourage the electrophysiology measures during fMRI to explore the impact of neural circuitry on behaviour.

There are inherent limitations of Looping Star which result from adaptations of the sequence for near-silent scanning, for example Dionisio-Parra et al. (2020) reported more signal dropout in Looping Star, compared to GRE-EPI, with a 15.9% signal dropout in grey matter which was related to the low temporal signal to noise ratio (SNR). It may be that the inability to map PPI neural correlates is associated with low SNR yet it should be emphasised that startle habituation neural circuitry was mapped. Future research directions for Looping Star will look to increase temporal SNR and demonstrate the application of a reliable, silent fMRI sequence.

There are several limitations in the current study. Firstly, the acoustic startle paradigm which was designed to explore two forms of startle plasticity may have affected startle habituation and PPI. A combination of pulse and PPI trials in the acoustic paradigm may produce a shift in baseline startle response. Although our findings are in line with McDowell et al. (2006) which used a pulse-only paradigm, it may be that combining stimuli type may have influenced short-term startle habituation. Moreover, startle responses to the pulse trials habituate, but responses to the prepulse trials do not thus, comparing startle magnitude on pulse trials to PPI trials will still show an inhibited startle response despite the smaller startle response to pulse trials (Blumenthal, 1997; Quednow et al., 2006). Finally, although sex effects and menstrual cycle phase were controlled in analyses, differences may have affected the findings, such as a larger cohort of females in this sample, compared to males, as previous literature has documented less PPI in women, compared to men (Swerdlow et al., 2016), and more PPI during the follicular phase, compared to luteal phase (Kumari et al., 2010).

## 5 Conclusion

This study measured acoustic short-term startle habituation, acoustic PPI, and related underlying neural activity in the young, healthy adults using simultaneous EMG acquisition and an innovative silent fMRI sequence, Looping Star (Wiesinger et al., 2019). Here, we observed a decrease in thalamic, striatal, insula, and brainstem regions during short-term startle habituation. Startle habituation was associated with altered cortical activity which may be consistent with RAS mediating the startle reflex response. Our findings in this healthy population may also encourage future clinical studies to probe clinical disorders such as Huntington’s disease (review, McDiarmid et al., 2017), which show startle habituation deficits and disturbances in RAS function (review, Garcia-Rill, 1997). The current study also examined acoustic PPI but did not observe significant neural activity and PPI was low in our sample. This may be associated with the MRI environment creating a floor effect which was not seen in our previous work using a 70 dB noise background. Further optimisation of acoustic PPI in the silent MRI environment in healthy participants is needed. These findings will pave future research using acoustic startle paradigms and combined EMG-fMRI for startle modulation research and will also inform sensory information processing mechanisms in healthy populations which, with further development, can be used as a blueprint for clinical research in disorders with aberrant PPI and startle habituation.

## Data availability statement

The datasets presented in this study can be found in online repositories. The names of the repository/repositories and accession number(s) can be found at: https://osf.io/j5vhp. The MRI data that support the findings of this study are available from LN upon reasonable request.

## Ethics statement

The studies involving humans were approved by Psychiatry, Nursing and Midwifery Research ethics committee at King’s College London. The studies were conducted in accordance with the local legislation and institutional requirements. The participants provided their written informed consent to participate in this study.

## Author contributions

LN: Conceptualization, Data curation, Formal analysis, Investigation, Methodology, Project administration, Writing – original draft, Writing – review & editing. OO'D: Formal analysis, Writing – original draft, Writing – review & editing. AS: Resources, Software, Writing – original draft, Writing – review & editing. FW: Resources, Software, Writing – original draft, Writing – review & editing. SH: Methodology, Resources, Writing – original draft, Writing – review & editing. SW: Conceptualization, Funding acquisition, Supervision, Writing – original draft, Writing – review & editing. VK: Conceptualization, Formal analysis, Supervision, Visualization, Writing – original draft, Writing – review & editing.

## Footnotes

^1^ http://www.fil.ion.ucl.ac.uk/spm
