# Supplementary material for: Investigating acoustic startle habituation and prepulse inhibition with silent functional MRI and electromyography in young, healthy adults
Source: Front Hum Neurosci. 2024 Aug 12;18:1436156. doi: 10.3389/fnhum.2024.1436156 (PMC11345142; doi:10.3389/fnhum.2024.1436156)
Supplement: Supplementary file 1 [file Data_Sheet_1.PDF]

Supplementary Material

1 EMG

1.1 EMG set-up

The EMG set-up was guided by the application note, “EMG signal processing during fMRI”, which can be found online (http://www.biopac.com/application-note).

Shorter MR-safe electrode leads are preferred to prevent wires from becoming twisted, thus we opted for 15cm leads.

1.2 EMG set-up

Gain is an adjustment of the hardware system to improve the SNR. Gain of 1000 is typically required for EMG studies, but due to use within the MRI scanner, which obscures the EMG signal, a higher gain was needed for the signal to be large enough. We opted to use a sampling rate of 10kHz. Here we used a higher sampling rate as this will characterise MRI noise during scanning.

1.3 EMG signal processing

According to Rose (2011), there are six stages in EMG signal processing: 1) raw signal amplification, 2) analogue band-pass filter, 3) analogue to digital conversion at a steady sampling rate, 4) rectify and digital low pass filter OR root mean square filter, 5) on, off time determination, and 6) time-frequency analysis. This approach was modified for the current study as EMG was captured within the MRI scanner and required additional signal processing. Typically, EMG studies are subject to motion artifacts, caused by head movement, and the environment, such as interference from power source. Within the MR environment, gradient artifacts are present, induced by the magnetic field gradients during scanning, and motion and environment artifacts are enhanced (Bullock, Jackson & Abbott, 2021).

The process was guided by Rose (2011), BIOPAC application note “EMG signal processing during fMRI”, EEG-silent fMRI research (Dionisio et al., 2018) and the basic principles of Looping Star, which differ in comparison to conventional sequences such as EPI:

1) The EMG signal, which is the difference in voltage between the two electrodes with the ground electrode, is acquired in real-time and recorded with AqKnowledge (BIOPAC Systems Inc.).
2) Band-pass filtering occurred during signal acquisition to remove low and high frequencies. Low frequencies are movement-related, whereas high frequencies are related to random noise. High frequencies are sensitive to aliasing, meaning the signal can be misidentified in error.
3) It is recommended that a high sampling rate is used to prevent aliasing. Rose (2011) suggested a sampling rate of five times the low-pass filter (500Hz * 5 = 25kHz) or more.
4) BIOPAC guidelines for filtering the EMG signal in MRI scanning were based on conventional scanning sequences, such as EPI. EPI has a distinctive signature in the frequency domain which can be easily identified for artifact removal. However, due to the different noise characteristics of Looping Star associated with differences in image acquisition, an alternative method was taken.

i. Firstly, the raw EMG signal for each participant was filtered using a comb band stop filter (determined as the maximum frequency from the power-spectral-density estimation for each participant on each run). The comb band stop filter removes electrical noise.
ii. Next, an average of all the cycles (1 cycle = TR = 2.62s) in the raw EMG signal was created and averaged over the entire sequence, starting from 0s.
iii. The averaged signal reflects the estimated effect of the MRI scanner on the EMG signal; thus, this averaged waveform was then subtracted from the raw EMG signal. This is known as average artifact subtraction, which is adapted to remove the gradient artifacts and identifies the artifact at each TR epoch.
iv. Finally, a low pass filter (30Hz) was applied to this new waveform, which was the EMG signal without the estimated MRI noise. The low pass filter removed high frequency responses.

5) The raw signal is transformed to the average rectified EMG. The combination of the low pass filter (30Hz) and rectifying the signal computes the linear envelope. We opted for this method, rather than the root square mean because this (root square mean) is more prone to estimation errors due to response outliers.
6) EMG data can now be scored after processing the raw EMG data.
    i. Prior to data scoring, EMG response to each trial was reviewed, and any trial with evidence of ongoing blinks before stimulus onset were excluded. Scoring criteria were identical to those reported previously (Kumari et al., 2008) and exclusion criteria from analysis has been presented earlier.

To explore the raw ASR amplitude on each trial, the mean voltage of the average rectified EMG signal from each trial onset for 100ms was used. The 100ms window is thought to reflect the reflex response, as a response outside of this window would not be related to the stimulus.
