# Supplementary material for: Investigating acoustic startle habituation and prepulse inhibition with silent functional MRI and electromyography in young, healthy adults
Source: Front Hum Neurosci. 2024 Aug 12;18:1436156. doi: 10.3389/fnhum.2024.1436156 (PMC11345142; doi:10.3389/fnhum.2024.1436156)
Supplement: Supplementary file 2 [file Image_1.PDF]

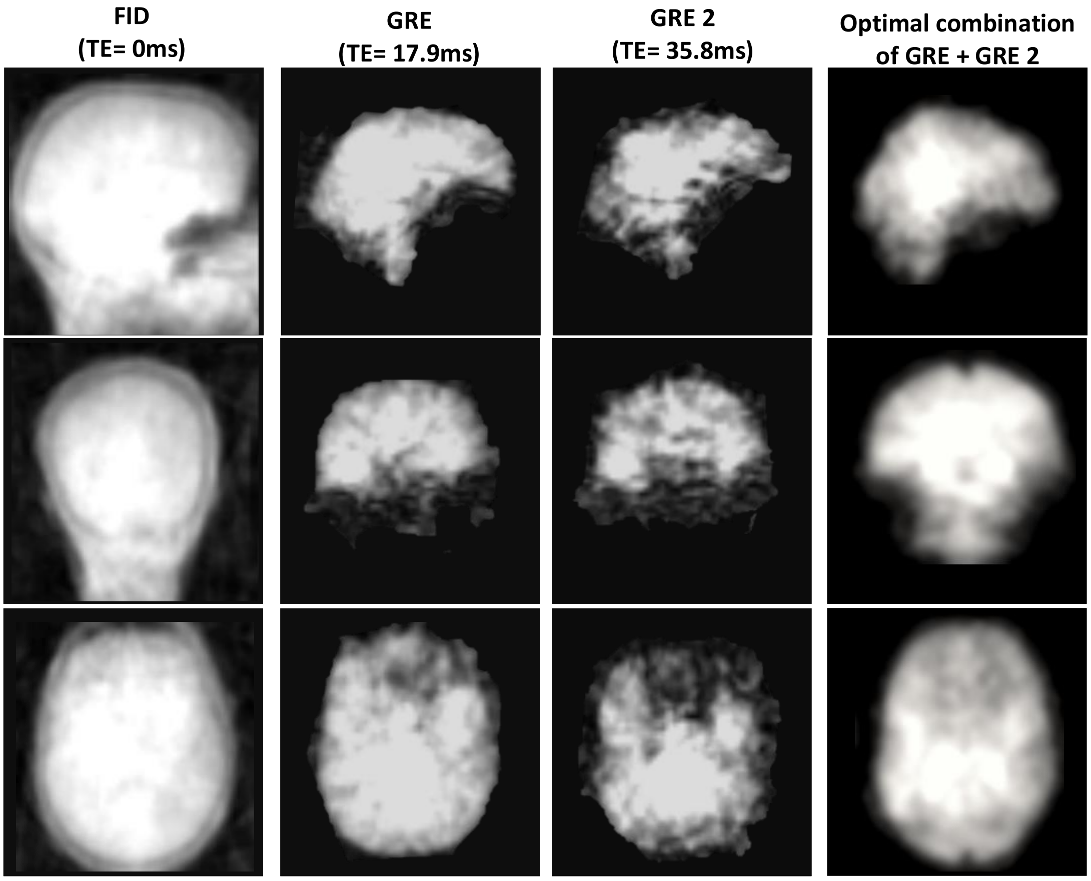

Supplementary Figure 1. Sagittal, coronal, and axial slices of free induction decay (FID), gradient echoes (GRE) (1 and 2), and optimal combination of gradient echoes of one participant.
