# Supplementary material for: Investigating acoustic startle habituation and prepulse inhibition with silent functional MRI and electromyography in young, healthy adults
Source: Front Hum Neurosci. 2024 Aug 12;18:1436156. doi: 10.3389/fnhum.2024.1436156 (PMC11345142; doi:10.3389/fnhum.2024.1436156)
Supplement: Supplementary file 3 [file Table_1.DOCX]

Supplementary Material

**Supplementary Table 1**. Montreal Neurological Institute (MNI) coordinates of the ROI masks in the startle habituation (brainstem mask + bilateral thalamus mask) and PPI (brainstem mask + PPI mask)

| Brain region | Hemisphere | MNI coordinates | | |
| --- | --- | --- | --- | --- |
|  |  | x | y | z |
| *ROI in the brainstem mask* | | | | |
| Pons |  | 0 | -26 | -30 |
| Midbrain |  | 0 | -20 | -7 |
| *Bilateral thalamus mask* | | | | |
| Thalamus | R | 13 | -18 | 8 |
|  | L | -11 | -18 | 8 |
| *ROI in the PPI mask* | | | | |
| Caudate | R | 15 | 12 | 9 |
|  | L | -11 | 11 | 9 |
| Hippocampus | R | 29 | -20 | -10 |
|  | L | -25 | -21 | -10 |
| Globus Pallidum | R | 21 | 0 | 0 |
|  | L | -18 | 0 | 0 |
| Insula | R | 39 | 6 | 2 |
|  | L | -35 | 7 | 3 |
| Thalamus | R | 13 | -18 | 8 |
|  | L | -11 | -18 | 8 |
| Putamen | R | 28 | 5 | 2 |
|  | L | -24 | 4 | 2 |
